# Identification of Floral Scent Profiles in Bearded Irises

**DOI:** 10.3390/molecules24091773

**Published:** 2019-05-07

**Authors:** Yuan Yuan, Ye Sun, Yanchun Zhao, Chungui Liu, Xiulan Chen, Fengtong Li, Jianzhong Bao

**Affiliations:** 1 Institute of Agricultural Sciences for Lixiahe Region in Jiangsu, Yangzhou 225007, Jiangsu Province, China; ocean1209@126.com (Y.Y.); sunye9999@126.com (Y.S.); horlcg@sina.com (C.L.); yzchxl@163.com (X.C.); 2 Civil Engineering Department, Yangzhou Polytechnic College, Yangzhou 225009, Jiangsu Province, China; zhaoyc083@163.com

**Keywords:** bearded iris, floral scent component, GC-MS, SPME

## Abstract

Bearded irises are ornamental plants with distinctive floral fragrance grown worldwide. To identify the floral scent profiles, twenty-seven accessions derived from three bearded iris, including *Iris. germanica*, *I. pumila* and *I. pallida* were used to investigate the composition and relative contents of floral scent components by headspace solid-phase microextraction (HS-SPME) and gas chromatography–mass spectrometry (GC-MS). A total of 219 floral scent components were detected in blooming flowers. The scent profile varied significantly among and within the three investigated species. Principal component analysis (PCA) indicated that terpenes, alcohols and esters contributed the most to the floral scent components and 1-caryophyllene, linalool, citronellol, methyl cinnamate, β-cedrene, thujopsene, methyl myristate, linalyl acetate, isosafrole, nerol, geraniol were identified as the major components. In a hierarchical cluster analysis, twenty-seven accessions could be clustered into six different groups, most of which had representative scent components such as linalool, citronellyl acetate, thujopsene, citronellol, methyl cinnamate and 1-caryophyllene. Our findings provide a theoretical reference for floral scent evaluation and breeding of bearded irises.

## 1. Introduction

Floral scent is a complex mixture of low molecular weight volatile secondary metabolite compounds released by plant flowers, which gives the flower its unique fragrance Floral scent not only plays an important role in plants’ reproductive process by attracting pollinators [1], but also enhances the aesthetic properties of ornamental plants [2]. Detailed studies on floral scent profiles have been carried out on many traditional aromatic plants, including orchids [3,4], *Osmanthus fragrans* [5], tree peony [6], *Lilium* [7], rose [8,9], *Prunus mume* [10], *Chimonanthus praecox* [11] and so forth. However, in recent years, ornamental plant researchers and breeders have paid more and more attention on some novel fragrant ornamental plants such as *Luculia pinceana* [12], *Hosta* [13], *Lagerstroemia indica* [14], whose floral fragrances are distinctive, so as to discover new floral scent components which could be applied in generating novel varieties.

Bearded iris is an *Iris* (Iridaceae) member that gains its name from the line of thick hairs that emerges from the throat of the flowers. These hairs form a long, furry caterpillar towards the back of the fall petals so as to guide pollinators towards the pollen [15]. The colors of the hairs could present different morphs among cultivars or species. Bearded irises are popular ornamental plants worldwide, widely used in gardens and flower borders, as potted plants, an even cut flowers and other production fields. In addition to the visible characteristics, bearded iris is one of the few groups in the *Iris* genus which produces a pleasant fragrance. As a result, bearded irises have a particularly high ornamental value in both the visual and olfactory sense. *I. germanica* has the greatest number of cultivated varieties in the bearded iris group. During the long evolution history of *I. germanica*, more than eight related species were introduced into hybridization breeding, which greatly improved the flower type, flower color, plant height, leaf color and floral fragrance ornamental traits of *I. germanica* varieties. Precisely because of this, *I. germanica* has become the most popular iris in garden use. So far, although great progress has been made in the studies on cultivation [16,17], propagation [18,19], genetics and breeding [20,21,22,23], chemical characterizations of the floral scent in *I. germanica* and other species of bearded iris have not been reported to date, which hinders the development and utilization for the varieties with different fragrances. 

In this work, we explore the floral scent profile variations of bearded irises, mainly in *I. germanica* wild species and cultivars (twenty-three accessions), as well as in *I. germanica’*s two related species *I. pallida* (two accessions) and *I. pumila* (two accessions) through headspace solid-phase microextraction coupled with gas chromatography-mass spectrometry. Firstly, we identify the specific floral scent volatile compounds in the three investigated bearded iris species and compare the scent compositions in the twenty-seven accessions with the expectation of encountering species-specific and/or cultivars-specific volatile components. Secondly, we clarify the most contributing components in the investigated bearded irises based on the scent component profiles through principle components analysis. Finally, we classify the twenty-seven accessions into different floral fragrance patterns according to their floral scent components by using hierarchical cluster analysis to clarify the origin of the distinctive fragrance of the flowers. The results would not only provide a theoretical reference for elucidation of the chemical mechanism of floral fragrance formation in bearded irises, but also lay a foundation for the fragrant varieties breeding in the future.

## 2. Results and Discussion

### 2.1. Identification of Floral Scent Components in Bearded Iris Accessions

The total ion chromatogram of scent components emitted from the flowers of twenty-seven accessions of three bearded iris species was shown in Figure S1. A total of 219 scent components in 10 categories including 42 terpenes, 19 alkanes, 11 aromatic compounds, 52 esters, 41 alcohols, 17 ketones, 21 aldehydes, nine ethers, four phenols, and three acids were identified by comparison with a known database (Table 1 and Table 2). The number of volatile components varied greatly among different species and/or cultivars, ranging from 34 (in *I. pumila* ‘Dash Away’) to 64 (in *I. germanica* ‘Rare Edition’). The most components (64, 61, 60) were detected in the three *I. germanica* cultivars ‘Rare Edition’, ‘Splashacata’ and ‘Rajah Brooke’, respectively, whereas the least components (34, 35 and 35, 35) were found in two *I. pumila* cultivars (‘Dash Away’ and ‘Brassie’), as well as two *I. germanica* cultivars (‘Spiced Custard’ and ‘Wanda Rezac’). In the two *I. pallida* cultivars (‘Dalmatica’ and ‘Albo Variegata’) 36 and 59 components were detected, respectively (Table 2).

In the twenty-seven accessions, twelve cultivars/species had as first dominant scent components terpenes whose relative areas could reach as high as 90.16% in *I. germanica* ‘Blessed Again’, 89.03% in *I. pallida* ‘Dalmatica’ and 74.62% in *I. germanica* ‘Amsterdam’. l-caryophyllene accounted for the highest relative content in the accessions of *I. germanica* ‘Casual Elegance’, *I. germanica* ‘Lenora Pearls’, *I. germanica* ‘Amsterdam’ and *I. pallida* ‘Dalmatica’, while β-cedrene in the *I. germanica* cultivars of ‘Blessed Again’, ‘Indian Chief’, ‘Summer Olympics’ and ‘Splashacata’. Besides, *I. germanica* cultivars of ‘Crown Princess’, ‘Abridged Version’, ‘Rare Edition’ and ‘Tulip Festavil’ were characterized by the highest level of limonene, thujopsene, tativene and α-pinene, respectively.

Five cultivars/species had as first dominant scent components esters whose relative areas could reach as high as 51.41% in *I. germanica* ‘Rajah Brooke’, 45.20% in *I. germanica* ‘Superstition’ and 39.29% in *I. germanica* wild species. Methyl cinnamate accounted for the highest relative content in the accessions of *I. germanica* wild species and *I. germanica* ‘Spiced Custard’ while methyl hexadecanoate, methyl caprate and citronellyl acetate in *I. germanica* ‘Rajah Brooke’, ‘Superstition’ and *I. pallida* ‘Albo Variegata’, respectively. Nine accessions, including seven *I. germanica* cultivars and two *I. pumila* cultivars, had as the first dominant scent components alcohols, of which the top three highest levels were detected in the *I. germanica* cultivars ‘Forever Blue’ (73.11%) and ‘Wanda Rezac’(48.95%), and *I. pumila* ‘Brassie’ (59.00%), mainly including the compounds linalool, citronellol, geraniol and nerolidol. *I. germanica* ‘Hold and Behold’ had the highest relative content of alkanes (23.83%), six of which were detected with the highest level corresponding to pentadecane (12.74%).

Differentiations in scent profiles among species or cultivars have been demonstrated in several ornamental plants. It is noteworthy that overall our samples exhibited more volatile complexity than *Chrysanthemum* (193 compounds across 39 accessions) [24], tree peony (146 compounds across 30 accessions) [6], *Narcissus* (84 compounds across nine accessions) [25], *Hosta* (70 compounds across 46 accessions) [13], *Silene* (60 compounds across 10 species) [26], *Phlox* (59 compounds across 22 accessions) [27] and so forth. In our study, α-longipinene, thujopsene, α-pinene, limonene, nerolidol, tetrahydrogeraniol, hexadecane, pentadecane, lauryl alcohol, l-caryophyllene, linalool, alloaromadendrene were detected in most of the accessions. Moreover, we found that the scent profile varied significantly among and within the three investigated species of bearded iris. For instance, although the two *I. pumila* cultivars both had alcohols as the most dominant scent components, only a few stereotypical scent components were observed in this individual species. An overlap in five of 66 compounds was detected between the two cultivars. Similar results were also observed in *I. pallida,* two cultivars of which had different terpene and ester dominant scent components, respectively. More interestingly, the highest relative content of esters was detected in *I. germanica* wild species, however in cultivars, the highest level of alcohols, terpenes and alkanes were detected as well, which indicates that the floral scent profile in *I. germanica* group has become more complex during the cultivation and domestication process. The related species such as *I. pallida* and *I. pumila* which have high contents of terpenes and alcohols might play an important role on the floral scent evolution of *I. germanica* cultivars group. The high complexity of floral scent components provides a wide range of genetic basis for improving and developing new bearded iris varieties with different types of fragrance in the future.

### 2.2. Principle Components Analysis of Floral Scent Components in Bearded Irises

**Figure 1:**
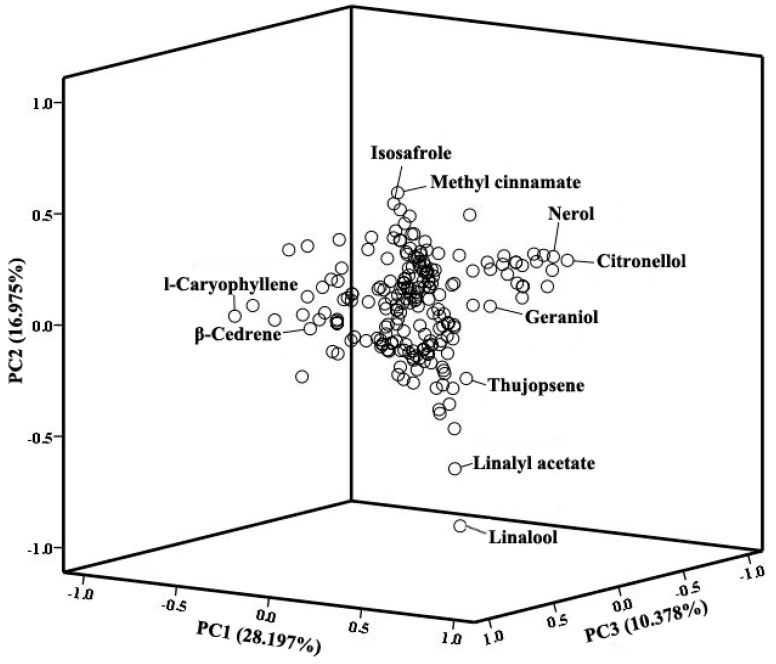
3D loading plot of Eigenvector load values for 219 floral scent components from PC1, PC2 and PC3.

Principle components analysis (PCA) was performed to simplify the multidimensional dataset based on the scent components profiles (Table S1). The results showed that the contribution rates of PC1-PC5 were 28.197%, 16.975%, 10.378, 8.528% and 7.463%, respectively, and the cumulative contribution rate reached 71.541%, which indicated that the first five PCs covered most of the scent component information of the investigated materials. As a result, we selected the component with the highest loading value in each of the first five PCs as the main factor, which showed that 1-caryophyllene, linalool, citronellol, methyl cinnamate and β-cedrene contributed the most to the scent components. Furthermore, as the cumulative contribution rate of the first three PCs reached 55.550%, we created a 3D loading plot to further explore the influence of each floral scent component on the differentiation of the twenty-seven accessions (Figure 1). The components with higher loading values explained more variance while those with low loading values explained less variance. The five compounds with the highest loading values in each of the first three PCs were extracted as the main scent components which contributed the most to each principal component. It could be seen that besides 1-caryophyllene, linalool, citronellol, methyl cinnamate and β-cedrene, thujopsene, methyl myristate, linalyl acetate, isosafrole, nerol and geraniol were also the representative floral scent components in the three bearded iris species.

**Figure 2:**
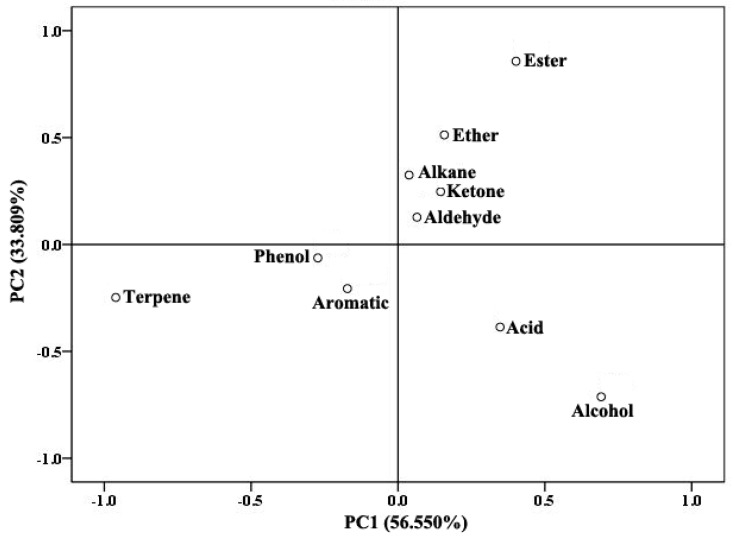
2D loading plot of eigenvector load values for 10 floral scent component categories from PC1 and PC2.

In addition, we also performed PCA analysis based on the 10 categories of floral scent components in the twenty-seven accessions (Figure 2). The first two PCs accounted for 90.359% of the total variance. PC1 explained 56.550%: terpene components were negative, while alcohols and esters were positive. PC2 (33.809% of the total variance) was positively related to alcohols and terpenes while negatively related to esters. The loading values of terpenes, alcohols and esters were the highest in both PCs indicating that the three categories of scent components contributed the most to the floral scent components in the three bearded iris species.

Bearded irises are not only ornamental plants, but also aromatic plants whose flowers are characterized by violet-like scent [28] and rhizomes constitute important sources of aromatic raw materials [29]. During a long drying time (3–5 years), the fats and oils in rhizomes undergo degradation and oxidation and then could release fragrant compounds called irones which could be used for iris essential oil production. Their scent was reported to resemble the one of violet flowers [28]. Besides, in *I. germanica* and *I. pallida,* an extract called resinoid with quite different odour (chocolate, woody, leathery and hay scent) could also be obtained from rhizomes, and eight isoflavones were identified in both resinoids [30]. However in our study, little irones and isoflavones were detected in the floral scent indicating that the composition of the floral scent emitted from flowers differs from that of the iris essential oil and resinoids extracted from rhizomes, and the secondary metabolites in iris flowers might be different from those in rhizomes. Similar results were also reported in two beardless species *I. pseudacorus* and *I. kerneriana* [31]. Moreover, the main floral volatiles detected in *I. pseudacorus* and *I. kerneriana* flowers, e.g. hexadecanoic acid, heptacosane, 6-methyl-5-hepten-2-one in *I. pseudacorus* and va-kessyl acetate, longipinene, decanoic acid, heptacosane, hexadecanoic acid, 6-methyl-5-hepten-2-one in *I. kerneriana* [31], were detected little in our study, which might indicate the different floral scent profiles between bearded and beardless irises. In our study, the floral scent volatiles could be classified into 10 categories including terpenes, alkanes, aromatic compounds, esters, alcohols, ketones, aldehydes, ethers, phenols and acids. Terpenes, alcohols, esters were the major components of floral scent according to PCA analysis. Similar classification was also reported in *Lilium* [7] and *Rosa rugosa* [8]. Our result provides a detailed evaluation for floral scent profile in bearded iris. However, the floral volatile organic compounds could be divided into terpenoids, phenylpropanoids/benzenoids, fatty acid derivatives, sulfur- and nitrogen-containing compounds based on major biosynthesis pathways [32]. Further investigations need to be carried out on the identification of the biosynthesis pathways in the differentially floral scent phenotypic accessions so as to mine the involved differentially expressed genes and analyze the relevant functions on floral scent regulation.

### 2.3. Hierarchical Cluster Analysis of Floral Scent Components in Bearded Irises

**Figure 3:**
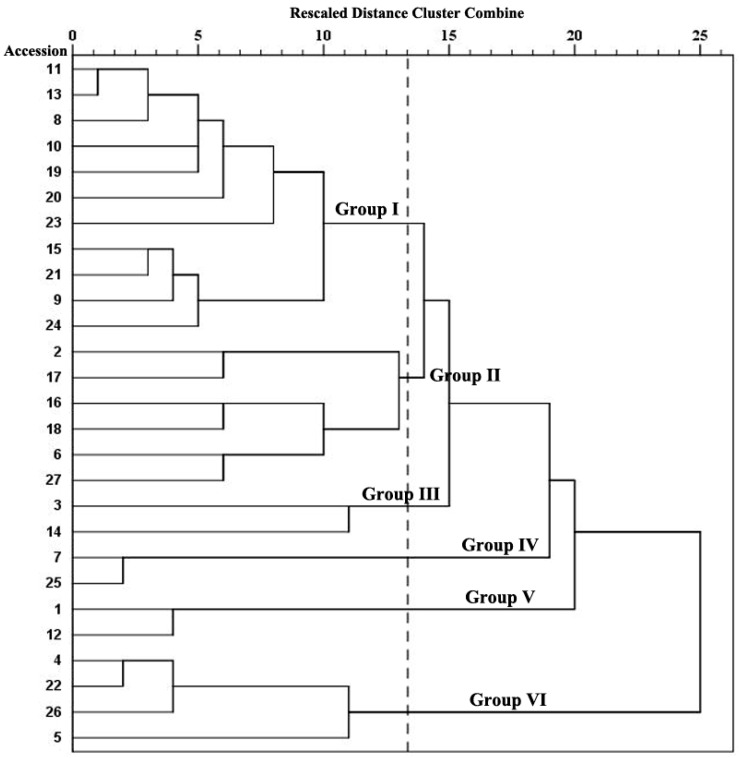
Hierarchical cluster dendrogram of twenty-seven bearded iris accessions.

To compare floral scent compositions among the twenty-seven accessions, we performed a hierarchical cluster analysis based on the relative contents of the 219 floral scent components. As is shown in Figure 3, by using Ward’s method for between-groups linkage and the squared Euclidean distance between clusters as a proximity measurement, twenty-seven accessions are clustered into six major groups. Eleven accessions including ten *I. germanica* cultivars and an *I. pumila* cultivar ‘Dash Away’ are clustered into Group I with higher relative content of linalool (12.22–34.61%). Six accessions including five *I. germanica* cultivars and an *I. pallida* cultivar ‘Albo Variegata’ are clustered into Group II with higher relative contents of methyl myristate (0–22.86%), methyl caprate (0–13.08%) or citronellyl acetate (0–15.90%). Group III contains accessions 3 (*I. germanica* ‘Swalli’) and 14 (*I. germanica* ‘Abridge Version’), which is with higher relative content of thujopsene (17.33%–21.92%). Group IV contains accessions 7 (*I. germanica* ‘Forever Blue’) and 25 (*I. pumila* ‘Brassie’), which is with higher relative content of citronellol (23.58–33.64%). Accessions 1 (*I. germanica* wild species) and 12 (*I. germanica* ‘Spiced Custard’) are in Group V with higher relative content of methyl cinnamate (22.59–34.16%). Finally, accessions 4 (*I. germanica* ‘Casual Elegance’), 5 (*I. germanica* ‘Blessed Again’), 22 (*I. germanica* ‘Amsterdam’) and 26 (*I. pallida* ‘Dalmatica’) are clustered into Group VI with higher relative content of 1-caryophyllene (24.64%–52.38%).

Surprisingly, hierarchical cluster analysis didn’t separate the three investigated species. The different accessions of *I. pumila* and *I. pallida* were clustering far apart: *I. pumila* in Groups I and IV and *I. pallida* Groups II and VI. Generally, the floral scent profile is species-specific, such as in the genus of *Magnolia* [33] and *Antirrhinum* [34]. However, certain environmental factors (including pollinators) as well as hybridized introgression could cause chemical change in the floral scent [33,35]. In our study, the three investigated species are all cultivated groups which have occurred wide gene recombination during the long history of selection and breeding. Linalool and linalyl acetate account for 56.60% of the floral scent in *I. pumila* ‘Dash Away’ while citronellol and nerol account for 48.83% in *I. pumila* ‘Brassie’. Thirty-six compounds are detected in *I. pallida* ‘Dalmatica’ while fifty-nine in *I. pallida* ‘Albo Variegata’. The large difference of the main floral scent components and composition might be responsible for the cluster results.

Floral scent is formed by the interaction of various volatile components. The component with higher scent value (content/olfactory threshold) could be considered as the characteristic floral scent component [36]. According to the result of hierarchical cluster analysis, most of the groups have representative scent components which are with higher relative contents. As previously reported, linalool, citronellyl acetate, thujopsene, citronellol, methyl cinnamate, 1-caryophyllene were responsible for the characteristic odor or fragrance profiles of sweet, lemon fruit-like, woody, rose flower-like, strawberry fruit-like and spicy [37,38], respectively. Therefore, the present study provides a more effective way for the olfactory evaluation and classification of the bearded irises with different floral fragrance, which would be an important theoretical reference for parents selecting and floral fragrance breeding in the future.

## 3. Materials and Methods

### 3.1. Plant Materials

A total of twenty-seven accessions including the wild species and twenty-two cultivars of *I. germanica*, two *I. pumila* cultivars, and two *I. pallida* cultivars were used in this study (Table 3). All the plant materials were grown under the same fertilizer and water management in the Iris Germplasm Resource Nursery (32°25’N; 119°23’E) in Institute of Agricultural Sciences for Lixiahe Region in Jiangsu, China.

### 3.2. Sample Collection

From April to May in 2018, the whole blooming flowers with fall petals fully expanded and the top of standard petals still closed (the second day after flower opening) were collected.

**Figure 4:**
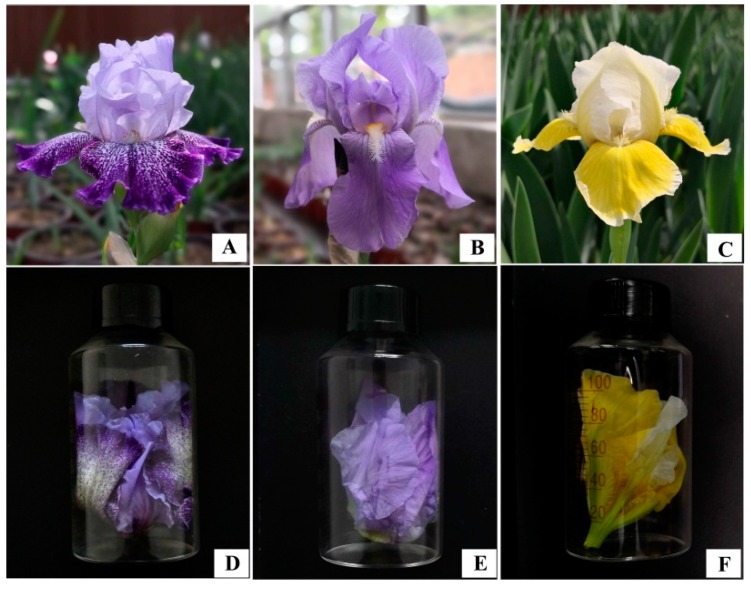
Photos of the representative accession for each of the three bearded iris species while flowering and inside 100 mL vials used for headspace extractions. (**A**,**D**) *I. germanica* ‘Splashacata’; (**B**,**E**) *I. pallida* ‘Dalmatica’; (**C**,**F**) *I. pumila* ‘Dash Away’.

Each cut flower was immediately placed into a 100 mL capped solid-phase microextraction vial (Figure 4), and then quickly transported to the laboratory for scent collection. All samples were taken within 60 min before floral scent sampling. These and subsequent procedures were repeated three times for each species and cultivar.

### 3.3. HS-SPME Analysis

HS-SPME analysis was performed by using a 75 μm carboxen-polydimethylsiloxane (CAR-PDMS) SPME fiber equipped with a manual SPME holder (Supelco, Bellefonte, PA, USA). Samples were equilibrated for 20 min at room temperature before analysis and the SPME fiber was conditioned at the gas chromatographic injection port for 40 min at 250 °C before the first volatile collection. After that, the fiber was inserted into the headspace of the capped vial with SPME holder to absorb volatile compounds for 40 min at 45 °C (water bath). The empty capped vial was used as the blank control.

### 3.4. GC-MS Analysis

When the extraction was complete, the fibers were withdrawn and inserted into the TRACE ISQ gas chromatography-mass spectrometry (Thermo Company, Waltham, MA, USA), and desorbed at 250 °C for 2 min. After that, the instrument was activated to collect data. TG-WAXMS column (60 m × 0.32 mm × 1.0 μm) was used for chromatographic analysis. The sample volume was 0.2 μL and helium (99.99%) was used as the carrier gas without splitting. The initial oven temperature was maintained at 50 °C for 1 min and then raised at 5 °C min^−1^ to 120 °C, then at 8 °C min^−1^ to 200 °C, and finally at 12 °C min^−1^ to 250 °C, maintained for 7 min. The temperatures of the injector, ion source were 250 °C and 200 °C, respectively. The ionization potential of mass selective detector was 70 eV with the 200 mu emission current and the scan mass range was 45–600 amu.

The volatile compounds formed different chromatographic peaks by the separating of gas chromatography and were qualitatively identified using the TF Xcalibur software by comparing the spectrometric data with those obtained from the NIST 08 mass spectral library and the Wiley library, combined with the manual resolution of mass spectra and confirmed by comparing the Kovat’s retention indices and relative reports from the literature [39]. Only results identified with positive and negative matching values of more than 800 (maximum is 1000) were selected and analyzed. All the compounds for each accession were analyzed under the same condition. The relative quantitative analysis was performed through peak normalization procedure. Peak areas were normalized as percentage and used to determine the relative amounts of the volatile compounds.

### 3.5. Statistical Analysis

The relative contents of all scent compounds emitted from the twenty-seven accessions were subjected to principal components analysis (PCA) by using a covariance matrix to calculate the Eigenvector load values and investigate the major floral scent components utilizing IBM SPSS version 20.0 (IBM, Armonk, NY, USA). Hierarchical cluster analysis was aslo performed to obtain consistent cluster results using Ward’s method, and the squared Euclidean distance between clusters was selected as the proximity measurement.

## 4. Conclusions

A total of 219 volatile compounds in floral scent were detected from fully open flowers of twenty-seven accessions in three bearded iris species *I. germanica*, *I. pumila* and *I. pallida*. There is considerable among/within species variation in floral scent components, which provides an extensive genetic basis for scent phenotype improving of bearded irises, especially *I. germanica*. The compositions of floral scent in bearded irises are different from those of essential oil and resinoid extracted from rhizomes, and also shows great difference compared to the beardless species *I. pseudacorus* and *I. kerneriana*. Although hierarchical cluster analysis couldn’t separate the three species, our finding that bearded irises could be clustered into different groups which had characteristic odor or fragrance profiles provides a reasonable and effective guidance for the olfactory evaluation and future breeding programs in bearded irises.

## Figures and Tables

**Table 1:** Floral scent compounds and relative contents emitted from twenty-seven bearded iris accessions.

Compound	Relative Content for Each Compound (%)																										
	1	2	3	4	5	6	7	8	9	10	11	12	13	14	15	16	17	18	19	20	21	22	23	24	25	26	27
Methyl cinnamate	34.16		0.20			7.71		0.34		13.54		23.59	1.67	10.48						0.22			0.92		2.43		0.23
Isosafrole	21.27							0.15		6.26		14.01	1.16							0.14			0.67		0.92		
Safrole	5.94																										
Farnesol	5.31				0.15						0.16		0.21					0.23			0.96		0.37			0.16	
α-Longipinene	3.24		0.13	0.91	7.40	0.75		0.15		0.14	0.25		1.29	0.19	0.11	1.09	1.09	2.51	2.86	0.44	0.23	1.23	0.17			4.66	3.34
Linalyl formate	3.03										0.10										0.74						
Thujopsene	2.45	1.90	18.33	1.70	0.11	0.31	0.53	0.51	10.11	2.30	1.57	0.18		21.92	5.95	3.82	0.22	0.31	0.37	0.25	5.36	0.24	1.18	17.58	*0.85*		4.44
Geranyl acetone	1.59					0.21					0.18	0.30	0.16	0.55		1.91		0.29		0.14			0.23	0.12			*0.65*
α-Pinene	1.56	3.51	5.70	0.42	0.46	0.48	0.81		2.19	1.69	0.83	11.10	1.15		11.97	1.23	4.02		1.03	0.61	10.71	3.69			2.76	0.46	0.24
Limonene	1.42	16.99	2.50		4.89		0.43		1.14	0.79	0.31	11.2	0.17		7.63	0.21	4.49		1.10		2.54	3.45			1.06	0.19	0.50
Nerolidol	1.23	0.60	1.57	0.31	0.13			18.15		2.78	12.63		11.07	0.38		0.61	0.61	0.19	0.10	0.60	0.62		22.52	1.46	0.56	1.61	1.72
Ttetrahydrogeraniol	1.05		0.35	1.38			0.30	0.32	0.11	1.26	0.14	0.76	0.49	0.25	0.82	19.04	0.26	3.58	0.35		2.72	0.28	0.69	4.12		0.32	
(-)-Verbenone	0.93		0.19						0.53	0.46		0.24			1.70			0.12									0.96
Menthol	0.82	0.10					0.76										0.17	0.58									
Isolimonene	0.72				1.29				0.11						2.22							1.84			0.44		
DL-Menthol	0.67																						0.17				
Isomenthol	0.62		0.44	0.13																							
Hexadecane	0.59	0.15	0.21	0.65	0.47	0.43			0.14	0.18		0.13		0.21	0.10	0.79	0.22	0.26	0.33	0.39	0.16			0.18		0.26	0.74
Safranal	0.55														1.29												
α-Phellandrene	0.47	1.11	0.14					0.49	0.10	0.61							0.14		0.64			0.54					
Butyl butyrate	0.45															0.23											
Citronellyl acetate	0.44					4.43	0.18												0.10		0.11		0.42		0.42		16.90
3-Methylheptane	0.43													0.13		0.11		0.12									
Farnesyl acetate	0.36															0.23					0.11						
Pentadecane	0.35	0.11	0.17	0.39	0.21	12.74	0.13	0.31	1.20	0.35	2.98	0.22	0.57	8.06		0.33	0.53	0.85	6.08	7.98	0.24	0.22	0.26	*0.28*	0.47		7.79
Butyl isobutyrate	0.29			0.10																							
Eugenol	0.27			3.88																	*0.27*						
Tridecane	0.26			0.39					0.19		0.41	0.11		0.74		0.11		0.10	0.21	3.05			0.46	0.48			0.31
Cineole	0.25		0.15						0.15	0.29		0.20			0.42		0.19				0.50				0.11	0.10	
1-Octanol	0.23									0.14					0.33	2.41								1.16			0.13
Tetradecane	0.22	0.12	0.10	0.20										0.13		0.38		0.28	0.18	0.42							0.62
1-Undecanol	0.21	0.20	0.15									0.17		0.19							0.11			0.16			0.11
Methyl oleate	0.21		0.20			0.42					0.24			2.50		0.19	0.11	0.86									
Camphene	0.21	0.42		0.14			0.15		0.70	1.28		0.50			2.56	0.15	1.35					0.83			0.88		*0.26*
*trans*-Dihydrocarveol	0.21		0.19				3.96	0.31	0.23	5.10														0.61			
β-Pinene	0.20	4.39	0.20		0.34			1.47	0.15	0.24		2.09		0.26	1.14		2.26	0.44	0.20		0.26	0.88			1.86		
Undecane	0.20			0.11					0.13					0.24	0.10	3.93			0.13					0.27			
β-Terpineol	0.17											0.17			0.33					3.78					0.13		
Cedrol	0.13			0.37	0.23							0.19				0.24		0.19		0.10	0.13					0.13	
Myrcene	0.13	4.38	0.75		0.29			0.31	0.30	0.34		1.08			1.38		2.08	0.76	0.37		0.91	0.80			2.87		0.11
Sabinene	0.13	0.45	0.27														0.85				0.26	0.22					
Methyl octanoate	0.12		0.35							0.10		0.14				0.59		1.10					0.18				0.12
Lauryl alcohol	0.12	0.20	0.11	0.12	0.20	2.42	0.18	0.27	0.12	0.44	0.13	0.24	1.74	0.70			0.22	0.50			0.24		0.62	0.53	0.59	0.36	0.32
Isoeugenol	0.12			0.15																							
Geranial	0.12		0.50				0.44																		1.99		
Terpinyl acetate	0.12	1.67	0.10							0.18					0.60								4.06	0.13			
7,8-Epoxy-α-ionone	0.11																							0.11			
Nonane	0.10	0.10		0.15													0.11										
Citronellyl propanoate	0.10					2.58	1.25												0.31	2.63	0.17		0.43		0.11		
3-Carene	0.10	1.49	0.98				1.32		0.10	1.45				0.25	0.3	0.22		0.33	0.49			0.38			9.23		0.64
l-Caryophyllene	0.10		0.10	45.44	24.64	0.38		0.27	0.25	7.46	9.64		17.11	0.71			1.14	5.72	0.12	19.6	0.50	37.41	0.90			54.38	10.71
Butyl hydroxy anisole	0.10															0.12											
Methyl myristate		22.86					2.55	2.67	7.87		2.10	9.72	5.40	3.30		1.93	20.25	2.29		0.83	7.76		5.56	2.85			0.70
Anisic aldehyde		5.30			0.24																			1.44			
Isoprene		3.30			0.13																	0.12					
Linalool		3.14	11.88	10.00		4.98	10.25	21.79	32.42	13.41	12.22		18.17		33.84	0.25	0.33		18.26	16.29	17.90	10.75	18.68	34.61	4.37		0.12
4-Carene		2.73			0.35				0.26			0.64			0.63		2.45					0.38					
Linalyl acetate		1.92	0.19			4.20	4.78		6.31	1.50	1.74		0.69		9.42				11.16	8.66	8.79	7.33		21.99			0.10
Geranyl phenylacetate		1.89													0.83		0.24										
Ethyl chrysanthemumate		1.67																						1.98			
Alloaromadendrene		1.54		1.19	2.80	1.95		0.87	2.66	1.53	5.58		0.39	11.75	1.20	5.21	0.13	0.38	5.50		5.64	5.47				0.65	0.16
Methyl laurinate		1.41							0.30			0.69	0.33			1.67	3.11	9.54			0.14			0.30			0.69
Sativene		1.33		1.08	0.19	2.02		0.20	0.76		0.53		0.13	0.46	0.69	7.80		1.63		0.58	1.64					0.41	
Methyl hexadecanoate		1.03					0.91	0.60	6.95		1.57	5.51	3.78	1.30		6.01	23.18	12.03		0.24	3.44		6.77	0.85			5.64
Methyl undecanoate		0.84						0.14					0.10	1.21		0.22	0.22	0.19			0.13		0.25	0.10			1.38
β-Cyclocitral		0.58																							0.69		
α-Guaiene		0.57		0.30	1.22	0.33		0.33						0.36	0.11	0.18		0.19			0.32					0.28	0.13
Ethylbenzene		0.53	0.50				0.29										0.86			0.19							
α-Terpinene		0.46	0.16		0.23							0.17					0.21					0.22			0.10		
*trans*-Carveol		0.32																									
α-Bulnesene		0.30			0.96	0.80		1.55		0.63	2.49			0.31		2.57		0.45	0.10			0.52				1.32	
(-)-4-Terpineol		0.24	0.26				0.13		0.18	0.13		0.60	0.11		0.23		0.59					0.41				0.15	0.12
Calarene		0.17		0.35	2.01								1.30	0.22			0.76		3.63		0.31	0.34					
1-Decanol		0.17												0.16	0.79						0.12			0.53			
Valencene		0.17			0.17	0.12		0.46	0.34		0.64			1.20	0.20	0.38		0.21	3.76		0.70						0.15
1-Methoxy-4-propylbenzene		0.16																									
Isocyclocitral		0.11																				0.10					
Methyl 10-undecenoate		0.11							0.16		0.24	0.50				0.57	0.30	0.47			0.24			0.13			0.24
Geraniol			22.64				5.70								0.25	0.63	1.91						2.11		2.87		1.99
2-Pentadecanone			5.91																								
α-Caryophyllene			4.82	0.41	0.67		0.12	1.03	0.14	0.28				16.7			0.18	1.63	0.11			3.95		0.34		0.71	
Nerol			3.43				12.4			0.15						1.85	0.18		1.33	0.54		3.90	0.13		23.25	0.55	0.21
Geranyl butyrate			1.03				0.13										1.42										
Neryl acetate			0.87				0.28										1.92						1.39		2.40		
2-Dodecanone			0.76																								
Methyl caprate			0.71										0.40			6.60		14.08		0.13				0.61			2.87
Isopulegol			0.62			0.10	0.12		0.37	0.66	0.13	0.70			0.80		0.16		0.33		0.69	1.07	0.33		0.22		
Phenylacetaldehyde			0.58					0.17																			
Methyl toluene			0.47	0.15															0.39								
α-Terpineol			0.40						0.46	0.83	0.62	0.63			1.09		0.95		0.36		0.64	0.81	1.19		0.36		
Citronellyl formate			0.37				0.12									0.16										0.17	
Tridecanal			0.36	0.11														0.36									
α-Santalol			0.35	0.47	0.70	0.12				0.13								0.25								0.10	0.10
Citronellol			0.27				33.64									0.55	0.26						2.14		25.58		
Diethyl carbonate			0.26																								
*cis*-Citral			0.20				0.12																		0.87		
Tricyclene		1.79	0.18				0.16								0.35	0.81			0.15		3.37				0.16		
Linalool propionate			0.18				0.27										0.13		0.11	0.10	5.10		2.27		0.16		
Undecanal			0.14									0.18		0.10													0.13
Cumin alcohol			0.14																								
Tridecanol			0.13			9.65				0.17				0.25		0.14			0.19	3.57	0.12						0.11
Aromadendrene			0.12	0.26	0.84			1.22						1.40		0.57				0.34	0.12						
Methyl benzoate			0.11		0.11																0.65					0.14	0.15
Tetradecanal			0.10	0.12	0.19	0.53	0.28	0.32	0.65	1.86	0.27		0.14	1.81		0.76	1.94	0.86					0.51			0.18	
α-Bisabolene				7.17	1.77	0.24		0.21	0.15		1.30		1.03					0.40	0.90	0.10		0.16				4.12	
α-Copaene				7.12							0.16		0.27				6.80		2.26	0.19							
Ketone				2.34																							0.17
β-Cedrene				1.53	29.36	5.56	0.13	16.94		9.89	19.29		13.89			3.41		12.92	11.07	1.63		8.66				7.10	3.20
α-Cubebene				1.05										0.81			1.15		0.65								
α-Cedrene				0.90	2.16	0.21		2.51	1.37	1.35	2.69		1.72				0.17	0.34	1.12	0.26		0.79				1.53	0.24
α-Bisabolol				0.79	0.39			0.18		0.12	0.27		0.17						0.12	1.54						0.10	
δ-Cadinene				0.68										0.10		0.19			0.29							10.64	
Patchouli alcohol				0.59																						0.11	
Propylbenzene				0.45				0.17					0.44														
α-Asarone				0.42																							
Heptadecane				0.36	0.41	7.68			0.18	0.19	0.11	0.16	0.13	1.05	0.22		0.28	0.10	0.42	0.38						0.17	0.5
Eugenol acetate				0.35																	0.19						
Piperonal				0.35						0.10								0.33		0.10			0.10				0.16
Viridiflorene				0.28				3.11			0.32		1.84	0.12		0.21			0.18				11.76				
2-Ethylhexanol				0.27																							
β-Asarone				0.18																							
Globulol				0.17				8.07			0.12						0.60										
α-Longifolene				0.17	4.45				0.15	1.47	2.54		0.17	0.17		0.39			0.22	0.23	1.99	1.18				2.28	
Caryophyllene oxide				0.16	0.10					0.12				0.23			0.17							0.30		0.12	
β-Santalol				0.16	0.37					0.13	0.17		0.26					0.17				0.18				0.34	
γ-Gurjunene				0.14	0.10	1.11		3.89			0.84			0.24	0.11	0.43		0.10			0.13						
1-Isoproyl-3-*tert-*butylbenzene				0.13													0.25										
(-)-Aristolene				0.11															0.23	1.26							
2-*tert*-Butyl-4-hydroxyanisole				0.10																							
2-Isopropyl-5-ketohexanal				0.10																							
β-Eudesmene					2.67			0.15												0.34	0.16					0.17	0.11
ρ-Diethylbenzene					0.60			0.36			0.64		0.26														
Benzyl formate					0.36																						
Phenylethyl alcohol					0.32							1.43										0.28		0.76			
γ-Terpinene					0.30						1.71				0.26		0.15					0.17					
Phenylacetic acid					0.22																						
Dolcymene					0.17	0.26		0.18			3.88		0.11						1.71	0.34		0.26					
Terpinolene					0.15	0.11											1.12					0.91					
2-Phenyl-1-propanal					0.11						0.11					0.24										0.23	
Isolongifolene					0.10				0.16																		
α-Thujone						13.41										0.43		0.11	1.47	2.58							6.1
2,2,4-Trimethylpentane						2.71																					
Myristyl alcohol						2.33	0.22	2.02		0.32				0.18			0.11		0.54	3.10							0.14
Cryptone						2.26														2.64							0.36
2,5-Dimethylhexane						0.17						0.13		0.49	0.32	6.47	0.18		0.22					0.21			
Dihydrocarvone						0.16													0.12								0.13
Methyl linoleate						0.16								1.21		0.16		0.32									
Methyl stearate						0.13								0.74			0.30	0.35									
Allyl hexanoate						0.11										0.46		0.18									
2,4-Dimethylpentane						0.10					0.10																
Cyclohexaneethanol							5.45												1.68								
Methyl nonanoate							2.00									0.34		1.39			0.11						0.19
Geranic acid							1.06																		0.37		
Menthol acetic ester							0.85																0.34		0.24		
Citronellyl butyrate							0.72														0.15						0.26
Crotonaldehyde							0.46																				
Carvomenthene							0.19																				0.10
Methyl salicylate							0.18																				
Benzyl benzoate							0.12							0.14													
Benzoic aldehyde							0.10																				
*m*-Isopropyl ethylbenzene								0.97											0.34								
Cinnamaldehyde								0.23		9.00		0.81															
*m*-Tolualdehyde								0.22					0.48														4.36
Amyl caprylate								0.16		0.11																	
Longicyclene								0.16					0.14						0.11		1.38	0.23					
Phenetole								0.12																			
Fenchole									13.59																0.86		
Pantolactone									1.79										1.35		2.01		0.75				
Methyl capronate									0.14																		
Eicosane									0.11						0.16			0.11								0.17	0.91
*m*-Diethylbenzene										0.42				0.99					0.72								
1-Nonanol										0.13						0.41											
*o*-Xylene										0.13																	
Thymol										0.12																	
Butyl caprylate										0.10	0.12																
Geranyl tiglate											2.31								1.49								
γ-Nonanolactone											0.99		0.21	0.15		0.48	0.23	0.12		1.58							
γ-Decalactone											0.90					0.26											
2,3,4-Trimethylpentane											0.43					0.60							0.69	0.16			
Tetrahydrofurfuryl acetate											0.31				0.58					1.42							
Nootkanone											0.13										0.15						
α-Cyclociral											0.11				0.10												
Hydroquinone Dimethyl												4.93						8.82			2.20						
Mesitylene													0.45														
Linalyl isovalerate													0.36														
Methyl acetate													0.21														
Methyl *m*-tolylketone													0.20						0.84								
2,2,5-Trimethylhexane														0.35						0.14							
Dodecane														0.13		0.52								0.19			
Octyl formate															0.33	0.27		0.20			0.36			0.23			
Cyclohexane																0.79		0.22									
2,6-Di-*tert*-butyl-*p*-cresol																0.58	0.11				0.13						0.1
γ-Valerolactone																0.42											
6-Decalactone																0.17								0.11			
Dihydro-α-ionone																0.13											
*trans*-2-Nonenal																	0.10										
*trans*-2-Pinanol																	0.38										0.27
2,2,4,4-Tetramethyl-3-pentanone																	0.20							0.27			
2-Methylpropanoic acetyl ester																		2.10	0.29	0.32							
2,3-Dimethylcyclohexanol																		0.39									
Decanal																		0.25				0.13					
β-Pinone																		0.15									
3-Methylpentane																		0.11						1.05			
β-Thujone																			2.58								
Isohexane																			1.12								
Neohexane																			0.82								
*p*-Methylbenzaldehyde																			0.24							0.51	
Pentanol																			0.18								
Citronellene																				2.71							
Pentadecanol																				0.19							
2-Ethyl-1-butanol																				0.19							
Cedryl acetate																					0.21						
Myristic acid																					0.10						
Isoamyl butyrate																								0.13			
Perillyl aldehyde																									0.12		
sogeraniol																									0.10		
Methyl anthranilate																											7.76
Piperitone																											3.01
3,5-Dimethyl-2-cyclohexen-1-one																											2.64

**Table 2:** Relative contents (%) and number of components for 10 floral scent compound categories emitted from twenty-seven bearded iris accessions.

Code	Terpene	Alkane	Aromatic	Ester	Alcohol	Aldehyde	Ketone	Ether	Phenol	Acid	Total
**1**	10.71 (12)	2.15 (7)		39.29 (10)	11.03 (13)	0.67 (2)	2.63 (3)	27.31 (3)	0.39 (2)		94.18 (52)
2	47 (19)	0.47 (4)	0.53 (1)	33.4 (9)	4.97 (8)	5.99 (3)		0.16 (1)			92.51 (45)
3	34.39 (14)	0.49 (3)	0.97 (2)	4.58 (12)	43.08 (17)	1.88 (6)	6.86 (3)				92.24 (57)
4	71.50 (22)	2.23 (7)	0.73 (3)	0.46 (2)	14.76 (12)	0.67 (4)	2.34 (1)	0.70 (3)	4.03 (2)		97.42 (56)
5	90.16 (29)	1.09 (3)	0.77 (2)	0.47 (2)	2.49 (8)	0.54 (3)				0.22 (1)	95.74 (48)
6	14.38 (14)	23.83 (6)	0.26 (1)	19.73 (8)	19.61 (6)	0.53 (1)	16.04 (4)				94.38 (40)
7	3.85 (9)	0.13 (1)	0.29 (1)	14.35 (14)	73.11 (12)	1.41 (5)				1.06 (1)	94.21 (43)
8	35.83 (20)	0.31 (1)	1.68 (4)	3.91 (5)	51.11 (8)	0.94 (4)		0.26 (2)			94.03 (44)
9	21.14 (19)	1.95 (6)		23.52 (7)	47.62 (9)	0.65 (1)	0.53 (1)				95.42 (43)
10	31.58 (17)	0.73 (3)	0.54 (2)	15.52 (6)	26.19 (17)	10.96 (3)	0.46 (1)	6.26 (1)	0.12 (1)		92.37 (51)
11	50.69 (17)	4.03 (5)	4.52 (2)	10.62 (11)	26.59 (10)	0.49 (3)	0.30 (2)				97.25 (50)
12	26.96 (8)	0.75 (5)		40.14 (6)	5.10 (10)	0.99 (2)	0.54 (2)	18.94 (2)			93.41 (35)
13	40.61 (14)	0.70 (2)	1.26 (4)	13.16(10)	32.20 (8)	0.62 (2)	0.36 (2)	1.16 (1)			90.08 (43)
14	57.41 (19)	11.52 (10)	0.99 (1)	21.03 (9)	2.12 (7)	1.90 (2)	0.55 (1)				95.53 (49)
15	36.81 (17)	0.90 (5)		11.76 (5)	38.87 (10)	1.39 (2)	1.70 (1)				91.44 (40)
16	28.85 (18)	14.03 (10)		20.96 (19)	26.14 (10)	1.00 (2)	2.47 (3)	0.12 (1)	0.58 (1)		94.16 (64)
17	30.90 (21)	1.31 (5)	1.11 (2)	51.41 (12)	7.05 (16)	2.03 (2)	0.20 (1)		0.11 (1)		94.13 (60)
18	28.32 (16)	2.15 (9)		45.20 (15)	6.06 (9)	1.79 (4)	0.67 (4)	8.82 (1)			93.02 (58)
19	37.45 (25)	9.52 (9)	3.16 (4)	14.81 (7)	23.45 (11)	0.24 (1)	5.00 (4)				93.63 (61)
20	28.54 (14)	12.36 (6)	0.53 (2)	17.13 (10)	29.90 (10)	0.10 (1)	5.37 (3)	0.14 (1)			93.06 (47)
21	36.84 (19)	0.40 (2)		30.41 (18)	24.75 (12)		0.15 (1)	2.20 (1)	0.40 (2)	0.10 (1)	95.25 (56)
22	74.62 (26)	0.22 (1)	0.26 (1)		17.69 (8)	0.22 (2)					93.02 (38)
23	14.00 (4)	1.42 (3)		30.68 (13)	48.95 (11)	0.62 (2)	0.23 (1)	0.67 (1)			96.57 (35)
24	18.22 (3)	2.81 (8)		29.43 (12)	43.93 (9)	1.44 (1)	0.50 (1)				96.33 (34)
25	24.22 (10)	0.47 (1)		5.76 (6)	59.00 (12)	3.68 (4)		0.92 (1)		0.37 (1)	94.42 (35)
26	89.03 (16)	0.60 (3)		0.14 (1)	4.04 (12)	1.09 (4)					94.89 (36)
27	24.32 (15)	10.87 (6)		37.24 (14)	5.34 (12)	4.66 (3)	14.02 (8)		0.10 (1)		96.54 (59)

**Table 3:** Species and cultivars of bearded irises used in this study.

Code	Accession
1	*I. germanica* wild species
2	*I. germanica* ‘Crown Princess’
3	*I. germanica* ‘Swahili’
4	*I. germanica* ‘Casual Elegance’
5	*I. germanica* ‘Blessed Again’
6	*I. germanica* ‘Hold and Behold’
7	*I. germanica* ‘Forever Blue’
8	*I. germanica* ‘Crinoline’
9	*I. germanica* ‘Immortality’
10	*I. germanica* ‘Indian chief’
11	*I. germanica* ‘Summer Olympics’
12	*I. germanica* ‘Spiced Custard’
13	*I. germanica* ‘Lenora Pearls’
14	*I. germanica* ‘Abridged Version’
15	*I. germanica* ‘Lent a Williamson’
16	*I. germanica* ‘Rare Edition’
17	*I. germanica* ‘Rajah Brooke’
18	*I. germanica* ‘Superstition’
19	*I. germanica* ‘Splashacata’
20	*I. germanica* ‘I’ ve Got Rhythm’
21	*I. germanica* ‘Tulip Festival’
22	*I. germanica* ‘Amsterdam’
23	*I. germanica* ‘Wanda Rezac’
24	*I. pumila* ‘Dash Away’
25	*I. pumila* ‘Brassie’
26	*I. pallida* ‘Dalmatica’
27	*I. pallida* ‘Albo Variegata’

## Supplementary Materials

The following are available online. Table S1: Loading matrix and Eigenvector values of the first 5 PCs, Figure S1: Total ionic chromatogram of scent components emitted from the flowers of twenty-seven accessions of three bearded iris species.
