# Supplementary material for: Identification of Floral Scent Profiles in Bearded Irises
Source: Molecules. 2019 May 7;24(9):1773. doi: 10.3390/molecules24091773 (PMC6540295; doi:10.3390/molecules24091773)
Supplement: Supplementary file 1 [file molecules-24-01773-s001.zip › Figure S1.pdf]

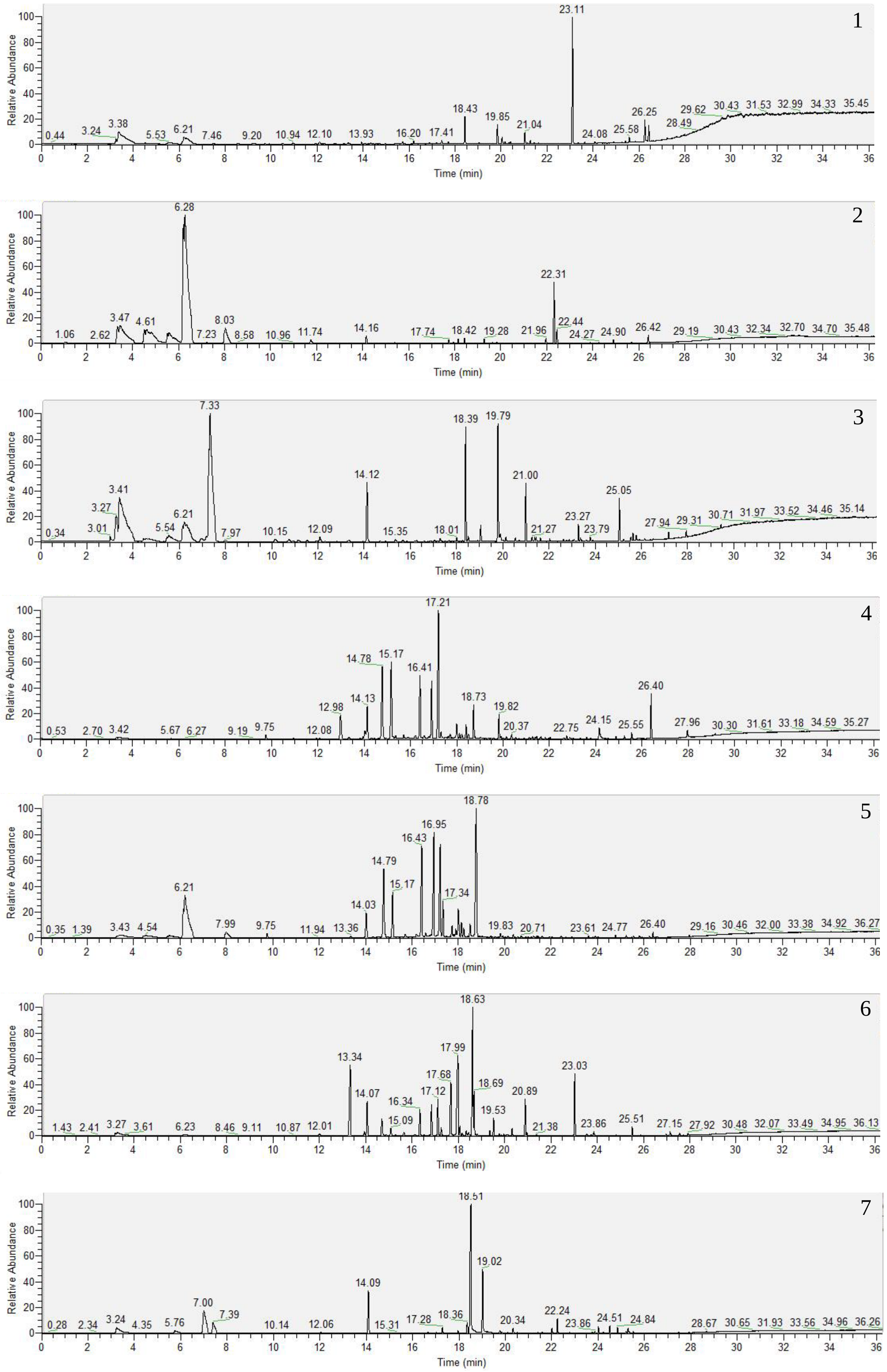
1
2
3
4
5
6
7
Relative Abundance
Time (min)
23.11
18.43
19.85
21.04
26.25
6.28
22.31
7.33
18.39
19.79
14.12
21.00
25.05
17.21
15.17
14.78
16.41
18.73
26.40
18.78
16.95
16.43
14.79
6.21
18.63
17.99
13.34
23.03
18.51
19.02
14.09
7.00

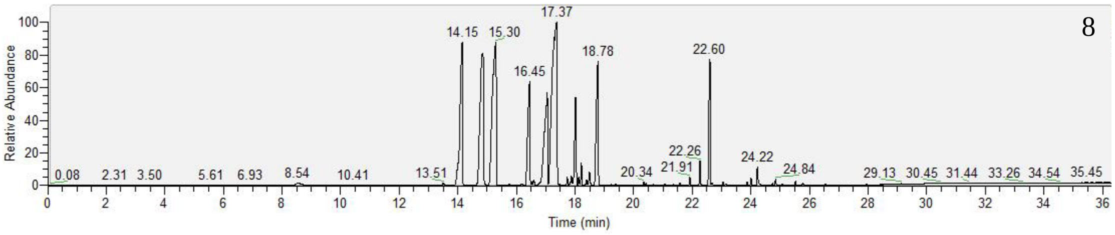
8
Relative Abundance
Time (min)
0.08 2.31 3.50 5.61 6.93 8.54 10.41 13.51 14.15 15.30 16.45 17.37 18.78 20.34 21.91 22.26 22.60 24.22 24.84 29.13 30.45 31.44 33.26 34.54 35.45

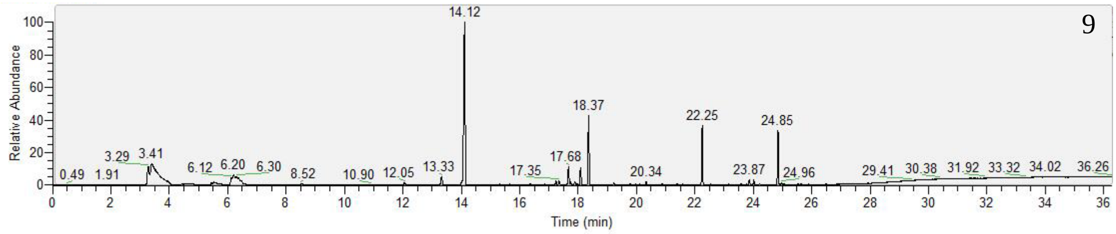
9
Relative Abundance
Time (min)
0.49 1.91 3.29 3.41 6.12 6.20 6.30 8.52 10.90 12.05 13.33 14.12 17.35 17.68 18.37 20.34 22.25 23.87 24.85 24.96 29.41 30.38 31.92 33.32 34.02 36.26

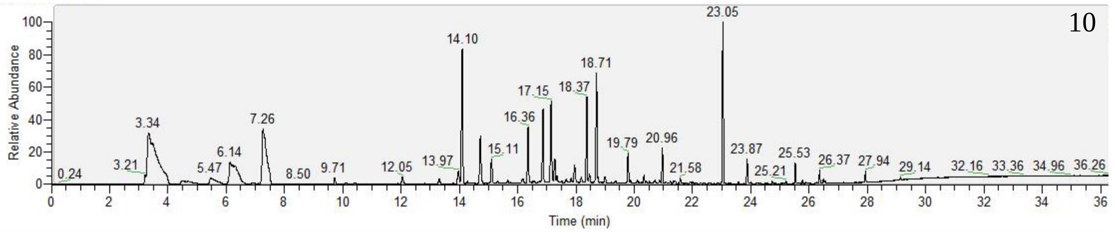
10
Relative Abundance
Time (min)
0.24 3.21 3.34 5.47 6.14 7.26 8.50 9.71 12.05 13.97 14.10 15.11 16.36 17.15 18.37 18.71 19.79 20.96 21.58 23.05 23.87 25.21 25.53 26.37 27.94 29.14 32.16 33.36 34.96 36.26

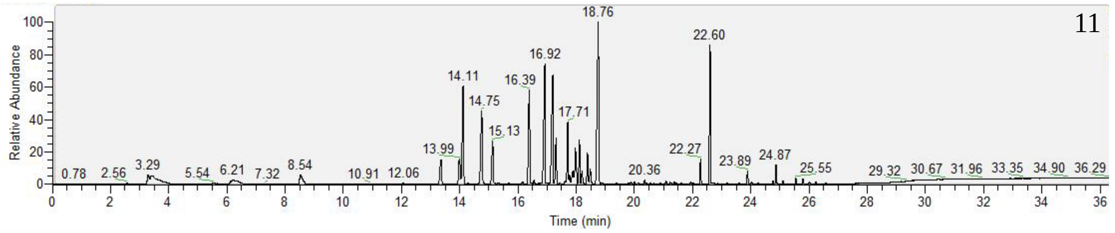
11
Relative Abundance
Time (min)
0.78 2.56 3.29 5.54 6.21 7.32 8.54 10.91 12.06 13.99 14.11 14.75 15.13 16.39 16.92 17.71 18.76 20.36 22.27 22.60 23.89 24.87 25.55 29.32 30.67 31.96 33.35 34.90 36.29

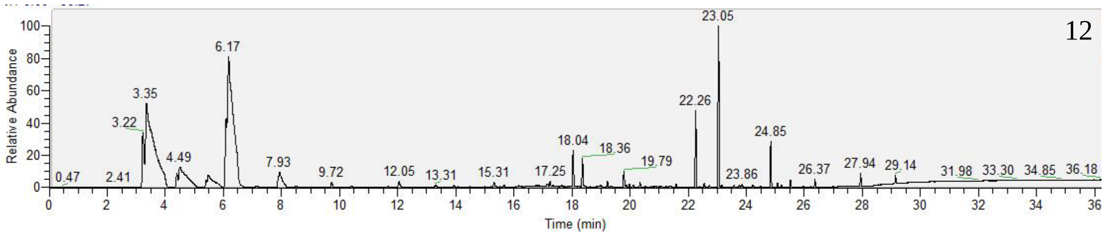
12
Relative Abundance
Time (min)
0.47 2.41 3.22 3.35 4.49 6.17 7.93 9.72 12.05 13.31 15.31 17.25 18.04 18.36 19.79 22.26 23.05 23.86 24.85 26.37 27.94 29.14 31.98 33.30 34.85 36.18

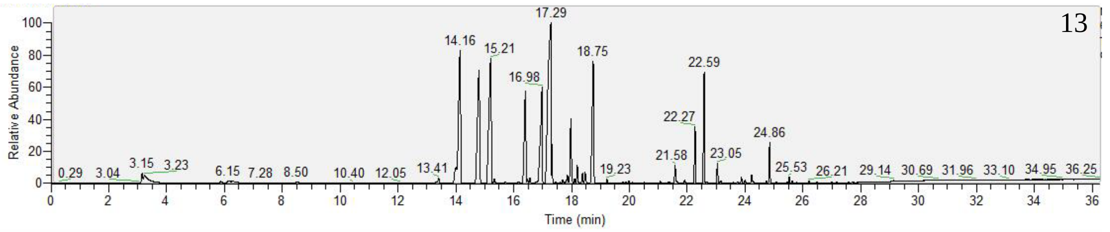
13
Relative Abundance
Time (min)
0.29 3.04 3.15 3.23 6.15 7.28 8.50 10.40 12.05 13.41 14.16 15.21 16.98 17.29 18.75 19.23 21.58 22.27 22.59 23.05 24.86 25.53 26.21 29.14 30.69 31.96 33.10 34.95 36.25

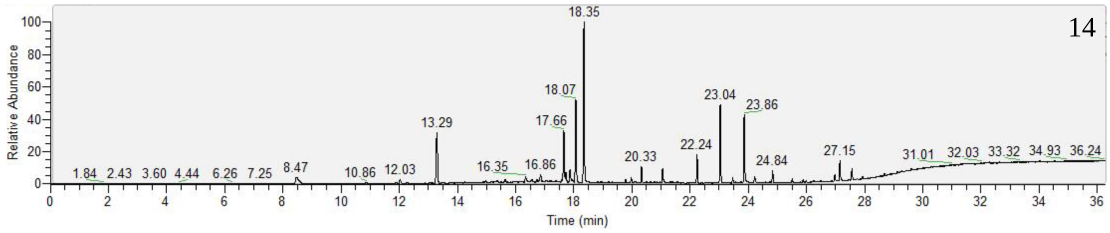
14
Relative Abundance
Time (min)
1.84 2.43 3.60 4.44 6.26 7.25 8.47 10.86 12.03 13.29 16.35 16.86 17.66 18.07 18.35 20.33 22.24 23.04 23.86 24.84 27.15 31.01 32.03 33.32 34.93 36.24

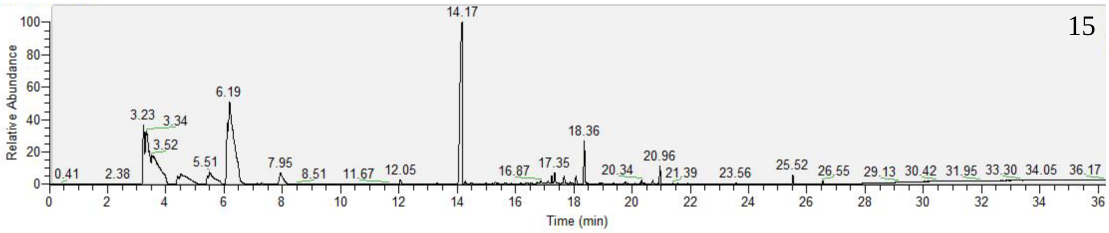
15
Relative Abundance
Time (min)
14.17
6.19
3.23
3.34
3.52
5.51
7.95
18.36
17.35
20.96
0.41 2.38 8.51 11.67 12.05 16.87 20.34 21.39 23.56 25.52 26.55 29.13 30.42 31.95 33.30 34.05 36.17

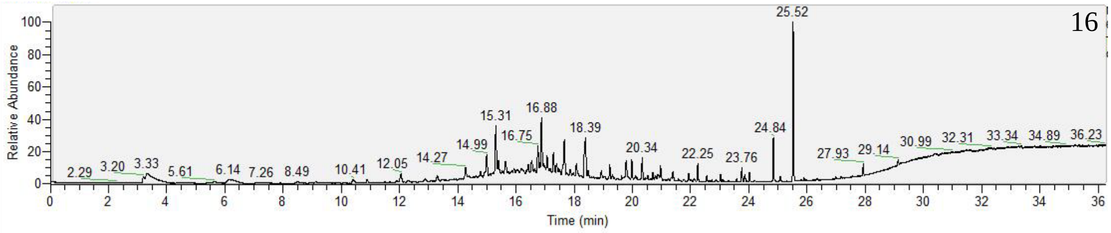
16
Relative Abundance
Time (min)
25.52
16.88
15.31
18.39
24.84
16.75
14.99
20.34
2.29 3.20 3.33 5.61 6.14 7.26 8.49 10.41 12.05 14.27 22.25 23.76 27.93 29.14 30.99 32.31 33.34 34.89 36.23

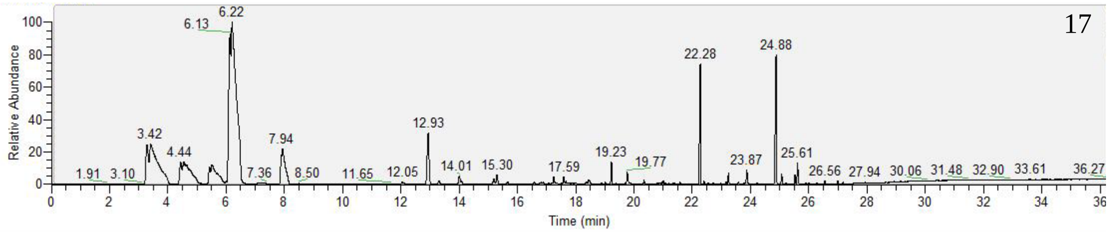
17
Relative Abundance
Time (min)
6.13 6.22
24.88
22.28
3.42
4.44
7.94
12.93
19.23
19.77
23.87
25.61
1.91 3.10 7.36 8.50 11.65 12.05 14.01 15.30 17.59 26.56 27.94 30.06 31.48 32.90 33.61 36.27

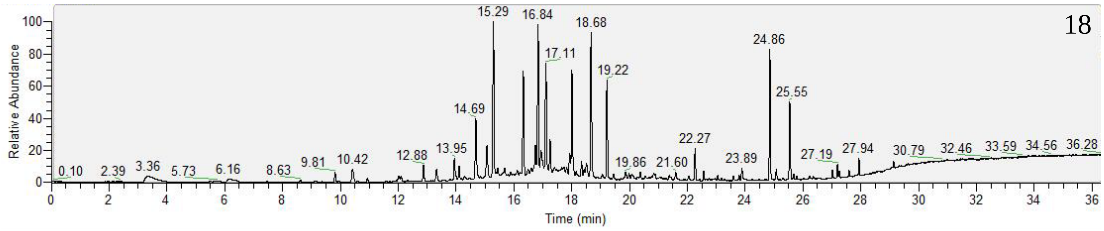
18
Relative Abundance
Time (min)
15.29
16.84
18.68
17.11
24.86
19.22
25.55
14.69
13.95
12.88
22.27
0.10 2.39 3.36 5.73 6.16 8.63 9.81 10.42 19.86 21.60 23.89 27.19 27.94 30.79 32.46 33.59 34.56 36.28

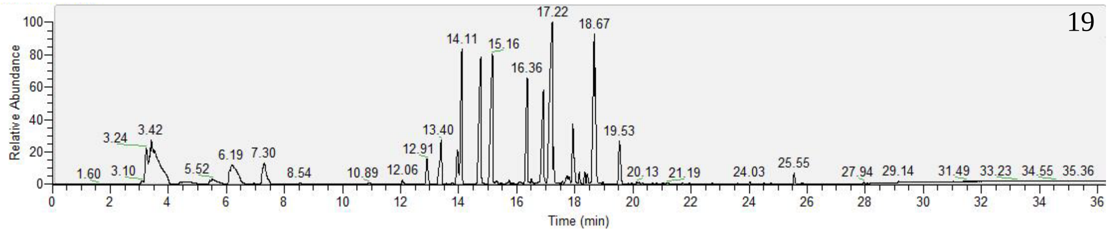
19
Relative Abundance
Time (min)
17.22
18.67
14.11
15.16
16.36
3.24
3.42
13.40
12.91
19.53
6.19
7.30
1.60 3.10 5.52 8.54 10.89 12.06 20.13 21.19 24.03 25.55 27.94 29.14 31.49 33.23 34.55 35.36

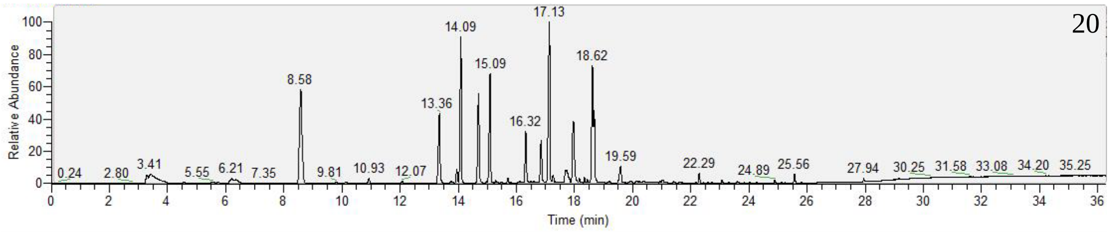
20
Relative Abundance
Time (min)
17.13
14.09
15.09
18.62
8.58
13.36
16.32
19.59
0.24 2.80 3.41 5.55 6.21 7.35 9.81 10.93 12.07 22.29 24.89 25.56 27.94 30.25 31.58 33.08 34.20 35.25

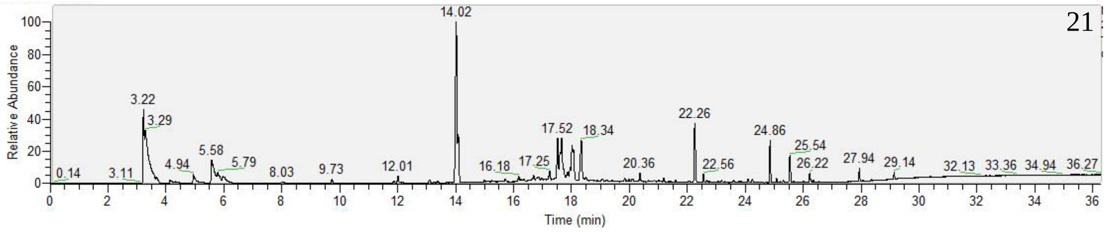
21
Relative Abundance
Time (min)
14.02
3.22
3.29
22.26
17.52
18.34
24.86
25.54
26.22
5.58
5.79
4.94
0.14 3.11 8.03 9.73 12.01 16.18 17.25 20.36 22.56 27.94 29.14 32.13 33.36 34.94 36.27

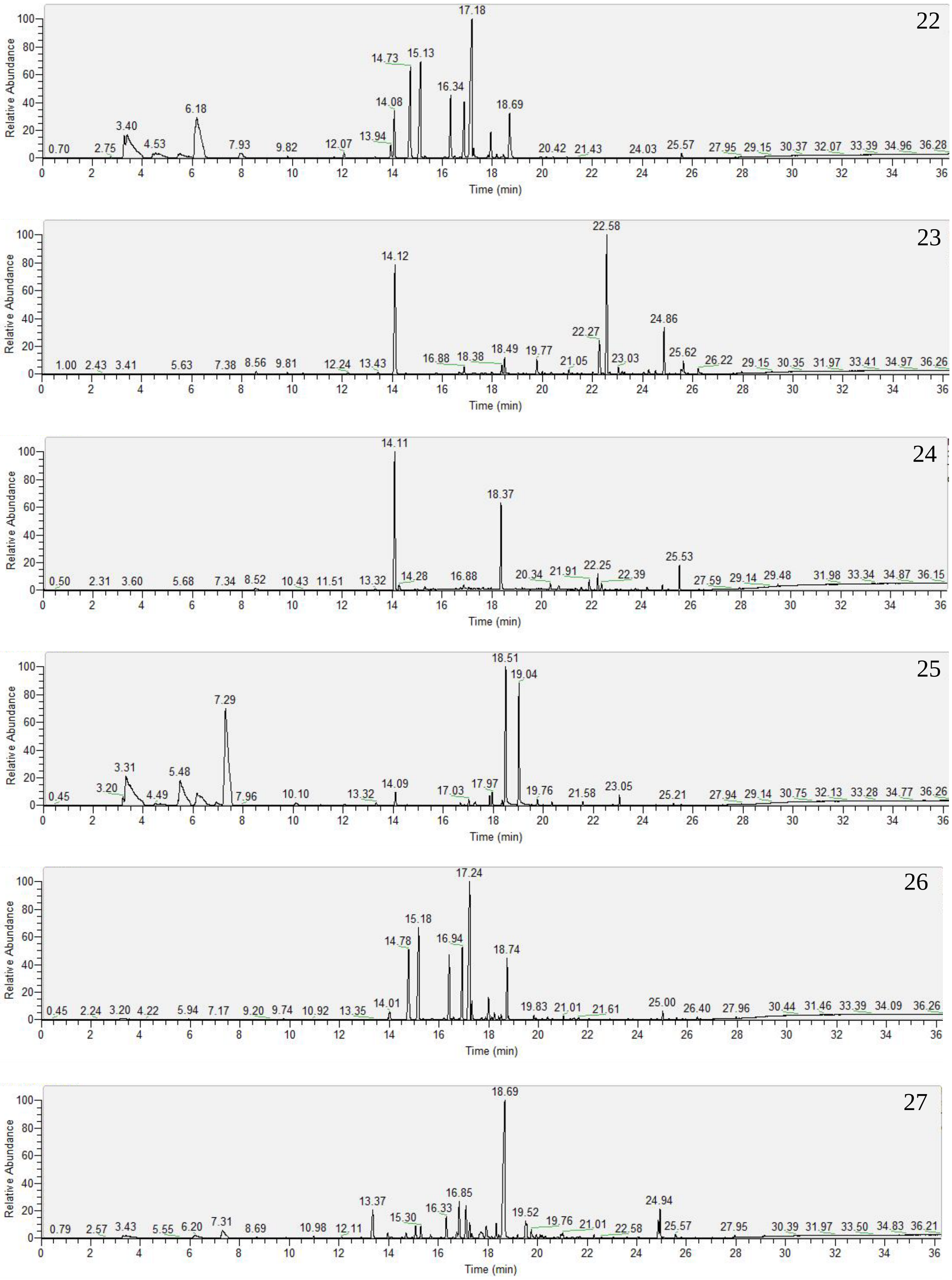

Figure S1. Total ionic chromatogram of scent components emitted from the flowers of twenty-seven accessions of three bearded iris species
