# Supplementary material for: Identification of Floral Scent Profiles in Bearded Irises
Source: Molecules. 2019 May 7;24(9):1773. doi: 10.3390/molecules24091773 (PMC6540295; doi:10.3390/molecules24091773)
Supplement: Supplementary file 1 [file molecules-24-01773-s001.zip › Table S1.pdf]

Table S1. Loading matrix and Eigenvector values of the first 5 PCs.

Variable	Principle component				
	**PC1 (28.197%)**	**PC2 (16.975%)**	**PC3 (10.378)**	**PC4 (8.528%)**	**PC5 (7.463%)**
Methyl cinnamate	1.653	5.130	3.322	-4.379	-2.454
Isosafrole	0.808	2.775	1.877	-2.467	-1.526
Safrole	0.127	0.490	0.334	-0.513	-0.337
Farnesol	0.113	0.409	0.316	-0.418	-0.296
α-Longipinene	-1.024	0.444	0.236	0.276	-0.322
Linalyl formate	0.083	0.232	0.179	-0.259	-0.152
Thujopsene	2.272	-1.413	0.746	-1.991	2.083
Geranyl acetone	0.071	0.232	0.113	-0.046	-0.042
α-Pinene	1.069	0.004	0.421	-0.831	1.060
Limonene	0.680	0.936	0.653	0.175	1.515
Nerolidol	0.350	-1.557	0.927	2.375	-2.867
Ttetrahydrogeraniol	0.397	0.308	0.113	0.591	0.484
(-) - verbenone	0.095	-0.013	0.098	-0.141	-0.014
Menthol	0.040	0.101	-0.045	-0.032	-0.042
Isolimonene	-0.092	-0.093	0.015	-0.133	-0.059
DL-Menthol	0.018	0.051	0.039	-0.052	-0.042
Isomenthol	0.014	0.046	0.028	-0.068	-0.017
Hexadecane	-0.057	0.080	0.052	-0.018	0.037
Safranal	0.055	-0.035	0.047	-0.090	-0.016
α-phellandrene	0.026	0.026	0.060	0.008	-0.004
Butyl butyrate	0.013	0.044	0.024	-0.030	-0.021
Citronellyl acetate	-0.112	0.563	-0.056	0.303	0.157
3-methylheptane	0.012	0.047	0.027	-0.029	-0.018
Farnesyl acetate	0.014	0.034	0.020	-0.023	-0.012
Pentadecane	0.141	0.305	0.360	0.015	-0.105
Butyl isobutyrate	-0.002	0.022	0.016	-0.029	-0.013
Eugenol	-0.312	-0.050	-0.010	-0.173	0.122
Tridecane	-0.046	-0.093	0.049	-0.102	0.032
Cineole	0.042	0.001	0.027	-0.049	0.019
1-Octanol	0.090	-0.019	0.026	0.011	0.058
Tetradecane	-0.015	0.041	0.011	0.009	0.029
1-Undecanol	0.028	0.028	0.025	-0.031	0.022
Methyl oleate	0.060	0.143	0.068	-0.007	0.068
Camphene	0.118	-0.056	-0.016	-0.094	0.115
trans-dihydrocarveol	0.161	0.018	-0.331	-0.234	-0.342
β-pinene	0.235	0.290	-0.030	0.218	0.348
Undecane	0.070	0.099	0.003	0.107	0.086
β-terpineol	-0.062	-0.093	0.005	-0.123	0.023
Cedrol	-0.049	0.029	0.014	-0.006	0.009
Myrcene	0.260	0.235	-0.219	0.174	0.423
Sabinene	0.027	0.043	0.011	0.048	0.113

Methyl octanoate	0.019	0.068	0.012	0.077	0.014
Lauryl alcohol	0.023	0.039	0.025	0.053	-0.107
Isoeugenol	-0.010	0.007	0.006	-0.016	-0.002
Geranial	0.073	0.070	-0.295	-0.068	-0.058
Terpinyl acetate	0.161	-0.088	0.057	0.160	0.008
7,8-epoxy-α-ionone	0.007	0.000	0.008	-0.014	-0.004
Nonane	-0.006	0.013	0.006	-0.004	0.015
Citronellyl propanoate	0.031	-0.014	-0.128	-0.074	-0.086
3-Carene	0.302	0.335	-1.228	-0.197	-0.209
l-Caryophyllene	-14.762	-1.656	-0.308	-2.087	1.237
Butyl hydroxy anisole	0.004	0.012	0.005	-0.004	-0.003
Methyl myristate	1.805	1.073	0.815	2.182	3.308
Anisic aldehyde	0.156	0.030	0.058	0.141	0.387
Isoprene	0.056	0.087	0.022	0.122	0.226
Linalool	3.520	-9.907	1.034	-1.863	-1.042
4-Carene	0.101	0.154	0.066	0.217	0.396
Linalyl acetate	1.308	-3.443	0.187	-1.602	0.645
Geranyl phenylacetate	0.073	0.008	0.024	0.057	0.161
Ethyl chrysanthemumate	0.112	-0.113	0.041	-0.025	0.155
Alloaromadendrene	-0.037	-0.061	0.492	0.149	0.250
Methyl laurinate	0.076	0.511	0.088	0.918	0.447
Sativene	0.078	0.200	0.046	0.387	0.307
Methyl hexadecanoate	0.969	1.183	0.515	2.704	2.182
Methyl undecanoate	0.043	0.095	0.032	0.074	0.131
β-cyclocitral	0.028	0.036	-0.079	0.007	0.021
α-guaiene	-0.088	0.035	0.029	0.083	0.000
Ethylbenzene	0.043	0.041	-0.037	0.054	0.117
α-Terpinene	-0.004	0.033	-0.003	0.024	0.045
trans-carveol	0.007	0.009	0.002	0.012	0.022
α-Bulnesene	-0.190	0.038	0.081	0.310	-0.215
(-)-4-Terpineol	0.007	0.039	0.016	-0.006	0.082
Calarene	-0.109	-0.088	0.066	0.189	-0.119
1-Decanol	0.057	-0.084	0.023	-0.047	0.040
Valencen	0.115	-0.107	0.082	0.093	-0.079
1-methoxy-4-propyl-Benzene	0.003	0.004	0.001	0.006	0.011
Isocyclocitral	-0.004	0.001	0.000	0.001	0.009
Methyl 10-undecenoate	0.035	0.058	0.031	0.065	0.054
Geraniol	0.896	0.054	-1.366	-0.405	0.652
2-pentadecanone	0.157	-0.041	-0.084	-0.130	0.189
α-Caryophyllene	0.073	0.449	0.246	-0.508	0.670
Nerol	0.671	0.717	-4.424	-0.784	-0.780
Geranyl butyrate	0.056	0.048	-0.026	0.068	0.145
Neryl acetate	0.151	0.108	-0.320	0.103	0.073
2-Dodecanone	0.020	-0.005	-0.011	-0.017	0.024

Methyl caprate	-0.002	0.631	0.011	1.191	0.264
Isopulegol	0.045	-0.061	0.020	-0.091	0.027
Phenylacetaldehyde	0.018	-0.010	-0.005	-0.004	0.006
Methyl toluene	0.006	-0.017	-0.005	-0.012	0.010
α-Terpineol	0.094	-0.078	0.044	0.012	0.029
Citronellyl formate	-0.004	0.004	-0.023	-0.009	0.016
Tridecanal	-0.002	0.007	-0.005	0.011	0.017
α-Santalol	-0.083	0.013	0.001	0.019	-0.008
Citronellol	1.554	1.039	-7.271	-1.014	-1.574
Diethyl carbonate	0.007	-0.002	-0.004	-0.006	0.008
cis-Citral	0.028	0.026	-0.123	-0.024	-0.021
Tricyclene	0.164	-0.025	0.003	0.066	0.250
Linalool propionate	0.203	-0.163	0.008	0.059	0.113
Undecanal	0.008	0.019	0.007	-0.010	0.011
Cumin alcohol	0.004	-0.001	-0.002	-0.003	0.004
Tridecanol	0.053	0.124	0.120	-0.074	-0.169
Aromadendrene	-0.020	0.015	0.052	0.066	-0.053
Methyl benzoate	-0.004	-0.010	0.003	0.000	0.026
Tetradecanal	0.085	0.144	0.084	0.152	0.096
α-Bisabolene	-1.193	-0.165	-0.029	-0.178	0.119
α-Copaene	-0.455	0.059	-0.004	0.203	0.714
Ketone	-0.198	-0.036	-0.017	-0.086	0.080
β-Cedrene	-3.313	-0.640	1.316	3.889	-4.374
α-cubebene	-0.042	0.035	0.017	0.020	0.137
α-Cedrene	-0.393	-0.255	0.171	0.295	-0.437
α-Bisabolol	-0.136	-0.067	0.010	-0.027	-0.018
δ-Cadinene	-1.243	0.020	-0.112	-0.299	0.278
Patchouli alcohol	-0.062	-0.010	-0.005	-0.026	0.022
propyl-Benzene	-0.044	-0.025	0.007	0.003	-0.012
α-Asarone	-0.035	-0.007	-0.003	-0.016	0.014
Heptadecane	0.050	0.193	0.131	0.002	-0.120
Eugenol acetate	-0.024	-0.010	-0.001	-0.014	0.018
Piperonal	-0.033	0.004	0.001	0.010	0.009
Viridiflorene	0.259	-0.439	0.172	0.631	-0.599
2-Ethylhexanol	-0.023	-0.005	-0.002	-0.010	0.009
β-Asarone	-0.015	-0.003	-0.001	-0.007	0.006
Globulol	0.111	-0.266	0.161	0.459	-0.546
α-Longifolene	-0.573	-0.045	0.105	0.194	-0.252
Caryophyllene oxide	-0.013	-0.010	0.011	-0.016	0.025
β-Santalol	-0.093	-0.004	0.008	0.020	-0.024
γ-Gurjunene	0.059	-0.113	0.107	0.255	-0.332
1-Isoproyl-3-tert-butylbenzene	-0.007	0.007	0.000	0.011	0.025
(-)-Aristolene	-0.033	-0.042	0.001	-0.031	0.008
2-tert-Butyl-4-hydroxyanisole	-0.008	-0.002	-0.001	-0.004	0.003

2-Isopropyl-5-ketohexanal	-0.008	-0.002	-0.001	-0.004	0.003
β-Eudesmene	-0.176	0.020	0.023	0.120	-0.117
ϱ-Diethylbenzene	-0.043	-0.022	0.025	0.083	-0.096
Benzyl formate	-0.021	0.005	0.003	0.017	-0.016
Phenylethyl alcohol	0.026	0.040	0.073	-0.078	0.010
γ-Terpinene	-0.037	-0.038	0.030	0.090	-0.087
Phenylacetic acid	-0.013	0.003	0.002	0.010	-0.010
Dolcymene	-0.050	-0.126	0.076	0.204	-0.273
Terpinolene	-0.048	0.029	0.001	0.057	0.094
2-fenyl-1-Propanal	-0.030	0.007	-0.001	0.013	0.000
Isolongifolene	0.000	-0.008	0.003	0.002	0.000
α-Thujon	0.059	0.356	0.140	0.117	-0.186
2,2,4-trimethylpentane	0.033	0.060	0.031	0.004	-0.058
Myristyl alcohol	0.007	-0.100	0.054	0.035	-0.189
Cryptone	-0.035	-0.013	0.024	-0.051	-0.020
2,5-Dimethylhexane	0.123	0.183	-0.006	0.225	0.160
Dihydro-carvone	0.002	0.003	0.002	0.004	-0.004
Methyl linoleate	0.026	0.060	0.025	-0.005	.039
Methyl stearate	.019	.050	.018	.023	0.046
Allyl hexanoate	0.006	0.021	0.000	0.030	0.008
2,4-Dimethylpentane	0.000	0.001	0.003	0.005	-0.007
Cyclohexaneethanol	0.173	0.002	-0.669	-0.083	-0.178
Methyl nonanoate	0.051	0.073	-0.246	0.070	-0.030
Geranic acid	0.037	0.021	-0.177	-0.027	-0.037
Menthol acetic ester	0.036	0.007	-0.133	-0.008	-0.037
Citronellyl butyrate	0.020	0.010	-0.089	-0.009	-0.008
Crotonaldehyde	0.012	0.004	-0.057	-0.008	-0.011
Carvomenthene	0.004	0.004	-0.024	-0.002	-0.003
Methyl salicylate	0.005	0.002	-0.022	-0.003	-0.004
Benzyl benzoate	0.006	0.006	-0.012	-0.006	0.001
Benzoic aldehyde	0.003	0.001	-0.012	-0.002	-0.002
m-Isopropyl ethyl benzene	0.019	-0.044	0.021	0.054	-0.080
Cinnamaldehyde	0.038	0.119	0.278	-0.239	-0.427
m-Tolualdehyde	-0.059	0.091	-0.005	0.109	0.047
Amyl caprylate	0.002	-0.005	0.006	0.006	-0.017
Longicyclene	0.022	-0.048	0.017	0.003	0.030
Phenetole	0.002	-0.004	0.002	0.006	-0.009
Fenchol	0.492	-0.765	0.097	-0.208	0.374
Pantolactone	0.153	-0.204	0.059	0.009	0.065
Methyl capronate	0.005	-0.008	0.002	-0.002	0.004
Eicosane	-0.021	0.011	-0.001	0.014	0.026
M-Diethylbenzene	0.031	0.017	0.035	-0.029	-0.007
1-nonanol	0.005	0.013	0.001	0.013	0.002
O-Xylene	0.000	0.001	0.003	-0.003	-0.006

Thymol	0.000	0.001	0.003	-0.003	-0.006
Butyl caprylate	-0.001	-0.001	0.004	0.003	-0.011
Geranyl tiglate	-0.004	-0.079	0.044	0.123	-0.161
γ-Nonanolactone	-0.039	-0.033	0.019	0.052	-0.012
γ-Decalactone	-0.007	-0.007	0.012	0.052	-0.042
2,3,4-trimethylpentane	0.025	-0.018	0.010	0.060	-0.026
Tetrahydrofurfuryl acetate	-0.016	-0.080	0.012	-0.038	0.002
Nootkanone	0.002	-0.005	0.003	0.006	-0.002
α-Cyclociral	0.002	-0.008	0.003	0.002	-0.005
Hydroquinone Dimethyl	0.122	0.573	0.263	0.386	0.129
Mesitylene	-0.009	-0.012	0.007	0.011	-0.014
Linalyl isovalerate	-0.008	-0.009	0.005	0.009	-0.012
Methyl acetate	-0.004	-0.005	0.003	0.005	-0.007
Methyl m-tolyl ketone	0.009	-0.029	0.009	0.014	-0.028
2,2,5-Trimethyl hexane	0.004	0.008	0.007	-0.013	0.012
Dodecane	0.017	0.005	0.003	0.008	0.018
Octyl formate	0.032	-0.033	0.010	0.001	0.027
Cyclohexane	0.009	0.030	-0.003	0.045	0.017
2,6-Di-tert-butyl-p-cresol	0.012	0.021	-0.002	0.031	0.027
γ-Valerolactone	0.005	0.012	-0.002	0.016	0.008
6-Decalactone	0.006	-0.004	0.001	0.002	0.006
Dihydro-α-ionone	0.002	0.004	-0.001	0.005	0.003
trans-2-Nonenal	0.002	0.004	0.000	0.007	0.008
trans-2-Pinanol	0.003	0.021	0.000	0.030	0.036
2,2,4,4-Tetramethyl-3-pentanone	0.014	-0.014	0.005	0.001	0.022
2-methyl-propanoic acetyl ester	-0.015	0.048	0.009	0.132	0.004
2,3-Dimethylcyclohexanol	-0.002	0.012	0.001	0.025	0.002
Decanal	-0.010	0.005	0.000	0.013	0.003
β-Pinone	-0.001	0.005	0.001	0.010	0.001
3-Methylpentane	0.040	-0.080	0.016	-0.038	0.021
β-Thujone	0.040	-0.072	0.017	0.027	-0.067
Isohexane	0.018	-0.031	0.007	0.012	-0.029
Neohexane	0.013	-0.023	0.005	0.008	-0.021
P-methyl benzaldehyde	-0.054	-0.005	-0.004	-0.011	0.006
Pentanol	0.003	-0.005	0.001	0.002	-0.005
Citronellene	-0.060	-0.073	0.000	-0.063	0.023
pentadecanol	-0.004	-0.005	0.000	-0.004	0.002
2-ethyl-1-butanol	-0.004	-0.005	0.000	-0.004	0.002
Cedryl acetate	0.005	-0.005	0.002	-0.001	0.007
Myristic acid	0.003	-0.002	0.001	0.000	0.003
Isoamyl butyrate	0.005	-0.010	0.002	-0.006	0.003
Perillyl aldehyde	0.003	0.004	-0.014	-0.002	-0.003
isogeraniol	0.002	0.003	-0.012	-0.002	-0.003
Methyl anthranilate	-0.093	0.197	-0.029	0.152	0.139

Piperitone	-0.036	0.077	-0.011	0.059	0.054
3,5-Dimethyl-2-cyclohexen-1-one	-0.032	0.067	-0.010	0.052	0.047
